# An ensemble-based 3D residual network for the classification of Alzheimer’s disease

**DOI:** 10.1371/journal.pone.0324520

**Published:** 2025-06-11

**Authors:** Xiaoli Yang, Jiayi Zhou, Chenchen Wang, Xiao Li, Jiawen Wang, Angchao Duan, Nuan Du

**Affiliations:** 1 School of Medical Technology and Engineering, Henan University of Science and Technology, Luoyang, China; 2 School of Basic Medicine and Forensic Medicine, Henan University of Science and Technology, Luoyang, China; University of South Florida, UNITED STATES OF AMERICA

## Abstract

Alzheimer’s disease (AD) is a common type of dementia, with mild cognitive impairment (MCI) being a key precursor. Early MCI diagnosis is crucial for slowing AD progression, but distinguishing MCI from normal controls (NC) is challenging due to subtle imaging differences. Furthermore, differentiating early MCI (EMCI) from late MCI (LMCI) is also important for interventions. This study proposes a deep learning-based approach using a weighted probability-based ensemble method to integrate results from three-dimensional residual networks (3D ResNet). (1) This study employs 3D ResNet-18, 3D ResNet-34, and 3D ResNet-50 architectures with the Convolutional Block Attention Module (CBAM). The attention mechanism enhances performance by helping the model focus on pertinent information. Data augmentation techniques are applied to address limited data and improve accuracy. (2) To overcome the limitation of the individual convolutional neural network (CNN), an ensemble learning method is adopted. The method assigns weights to each 3D CNN model based on prediction accuracy and integrates them to obtain the final result. Our method achieves accuracy of 94.87%, 92.31%, 95.49%, and 95.97% for MCI vs. NC, MCI vs. AD, EMCI vs. LMCI, and NC vs. EMCI vs. LMCI vs. AD, respectively. The results demonstrate the effectiveness of our method for AD diagnosis.

## Introduction

Alzheimer’s disease (AD) is an irreversible, progressive neurodegenerative disease and one of the most common causes of dementia in the elderly. As the disease progresses, patients experience a decline in cognitive abilities and daily functioning, which has a profound impact on their quality of life and that of their family members. Early diagnosis is crucial as it can help slow down the progression of the disease and improve the quality of life [1]. Mild cognitive impairment (MCI) represents a condition that exists between AD and the normal age-related cognitive decline in normal controls (NC). MCI is further classified into early MCI (EMCI) and late MCI (LMCI), with EMCI representing a milder form of cognitive impairment compared to LMCI. During the MCI stage, patients experience a slower decline in cognitive functioning, and in the majority of cases, the ability to perform activities of daily living remains largely unimpaired. However, MCI is considered a high-risk state for progression to AD, with approximately 10% to 15% of patients with MCI progressing to AD each year [2]. Therefore, it is essential to closely monitor individuals who are already in the MCI stage for changes in their cognitive functional status. Early intervention and treatment for them may help to delay or prevent the progression of MCI to AD [3].

In recent years, studies related to AD have demonstrated that neuroimaging techniques, such as magnetic resonance imaging (MRI) and positron emission tomography (PET), are more effective than traditional clinical assessments and psychological tests in diagnosing AD [4]. In particular, structural magnetic resonance imaging (sMRI), a widely used neuroimaging analysis method [5], plays an important role in the diagnosis of AD. As technology advances, the integration of neuroimaging techniques with computer-aided diagnosis has gained prominence in the classification and prediction of AD. For example, machine learning algorithms can analyze neuroimaging data to extract and interpret valuable information, thereby enhancing the accuracy of diagnoses and assessments [6].

Traditional machine learning algorithms usually require manual design and selection of features, which can be both time-consuming and challenging when dealing with complex neuroimaging data. In contrast, deep learning methods can automatically learn and extract high-level features from the data, capturing its intricate relationships more effectively. Consequently, deep learning techniques are becoming a more mainstream and effective option for medical image analysis. Convolutional neural networks (CNN) in deep learning have significant advantages in image processing tasks and are becoming more widely adopted [7]. Deep learning has been demonstrated exceptional performance in medical image analysis [8]. It also shows considerable potential in the classification and prediction of AD. In 2013, Suk et al. [9] explored the potential of deep learning in AD classification, proposing a deep learning-based feature representation using a stacked auto-encoder. This method revealed nonlinear relationships among features and improved classification accuracy. With the development of deep learning algorithms, more complex CNN architectures have been introduced into the research on AD, such as GoogleNet [10], VGGNet [11], and ResNet [12]. By introducing the residual learning mechanism, ResNet effectively solves the problems of gradient vanishing and gradient explosion of traditional CNNs during the training process. This advancement enables the network to learn complex feature representations more deeply and efficiently, enhancing the performance and training efficiency of deep learning models.

In the field of deep learning, two-dimensional (2D) images are widely used, especially in various applications related to computer vision and image processing. Some researchers have used 2D slices extracted from 3D MRI images to classify AD. For example, Xu et al. [13] introduced a selective kernel network and channel shuffle in ResNet and proposed an enhanced ResNet to classify AD. Prakash et al. [14] used whole slide 2D images to perform the classification tasks. They employed transfer learning with three models, ResNet-101, ResNet-50, and ResNet-18, and evaluated their performance in detecting AD. However, 2D images have certain limitations, as they are unable to directly convey 3D information or rotational transformations of objects. This may restrict the capabilities of deep learning models in certain scenarios. In the classification of AD, neuroimaging data is crucial, yet 2D images fail to fully utilize the volumetric information of brain imaging. To more accurately analyze and recognize brain information, researchers have introduced 3D CNNs. For instance, Frimpong et al. [15] proposed an AD classification model based on 3D CNN multilayer perceptron (MLP). The model uses an attention mechanism to automatically extract relevant features in the images and generate probability maps, which are then input to the MLP classifier. Zhang et al. [16] proposed a computer-aided method for early classification prediction of AD by introducing an explainable 3D residual attention deep neural network for end-to-end learning from sMRI scans. Wen et al. [17] compared numerous CNN-based research methods for AD classification and concluded that the performance of the different 3D methods was similar, while the performance of the 2D slicing methods was inferior. Unlike 2D CNNs, 3D CNNs process volumetric data, effectively capturing the spatial structure and relationships of objects from different angles. 3D CNN not only enhances the model’s ability to interpret complex data but also improves the accuracy and reliability of AD classification [18].

Input images for 3D CNNs can be categorized into three types: 3D whole-brain images, 3D image patches, and 3D regions of interest (ROI). Methods based on image patches may be inadequate for capturing the global features and structural context of the entire brain, potentially resulting in the overlooking of critical information dispersed across various regions. The performance of the ROI-based methods is relatively improved, but it is significantly influenced by the extraction and segmentation techniques. Whole-brain images require more computational resources and sophisticated data processing techniques, but they offer the most comprehensive and integrated information about brain structure and function [19].

The availability of medical image data is limited, which can pose certain challenges to classification tasks. In addition to advancements in network architectures, ensemble learning has been introduced to address these challenges. Tanveer et al. [20] proposed a model that combines ensemble learning with deep learning and employs transfer learning for AD classification, ultimately achieving better classification results. Zhang et al. [21] proposed a method combining 3D CNN with ensemble learning to improve the accuracy of AD classification. Furthermore, a data denoising module was proposed to reduce the boundary noise. Experimental results show that the model effectively improves the training speed of the neural network. Grover et al. [22] proposed an ensemble-based transfer learning method. It uses simple averaging ensemble and weighted averaging ensemble methods to extract superior sparse patterns and features from MRI images. An et al. [23] proposed a three-layer deep ensemble learning framework, including a voting layer, a stacking layer, and an optimizing layer. The proposed architecture demonstrated superior performance in AD classification compared to six other representative ensemble learning methods referenced in their paper.

To address the complexities and challenges in the classification of MCI, this study proposes an ensemble learning framework that integrates three distinct 3D ResNet models. Each base model is augmented with the Convolutional Block Attention Module (CBAM) to prioritize discriminative brain regions in 3D sMRI data, while data augmentation strategies are applied to mitigate the impact of limited data. The proposed method integrates predictions from these models using a weighted probability-based fusion strategy, which dynamically assigns weights based on the performance of each individual model. This study implements three binary classification tasks: AD vs. MCI, NC vs. MCI, EMCI vs. LMCI, and a multiclass classification task: AD vs. EMCI vs. LMCI vs. NC. The primary contributions of this study are as follows:

(1) Improved model design through 3D ResNets with CBAM, which enhances feature extraction by incorporating attention mechanisms alongside deep learning, enabling precise identification of brain changes in 3D MRI scans.
(2) The introduction of a weighted probability-based ensemble method to combine the classification results from three distinct 3D ResNet models. This method shows superior performance compared to using a single model, overcoming the limitations of individual CNNs. The proposed ensemble approach improves the accuracy of AD diagnosis and enhances the model's ability to distinguish between subtle cognitive stages such as early and late MCI.

The remainder of this paper is structured as follows:

The Materials and methods section explains the research data and methods in this study. The Results section presents the results of this study. The Discussion section discusses the experimental findings. Finally, the conclusion of this paper is provided.

## Materials and methods

**Fig 1:**
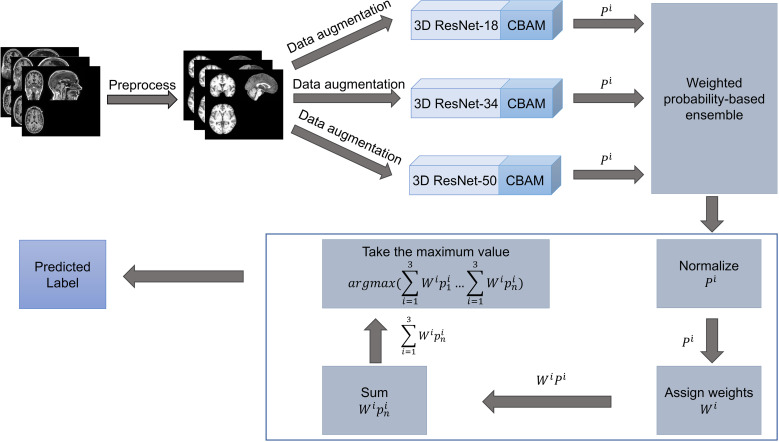
Workflow. *P*^*i*^ denotes predicted probabilities of the *i*-th model, *W*^*i*^ denotes the weight of the *i*-th model. *n* denotes the number of classes.

This section provides a detailed description of the data as well as the methods used in this study. The overall flow of the study is given in Fig 1. Initially, the 3D sMRI data undergo preprocessing. Subsequently, the data-augmented images are input into three classification networks for training. Finally, the classification results from the three networks are aggregated to produce final results.

### Materials

#### Data acquisition.

The data used in this study are sourced from the Alzheimer’s Disease Neuroimaging Initiative (ADNI) database (http://adni.loni.usc.edu/). The ADNI database is a widely utilized resource on a global scale, containing various forms of data, including sMRI, functional MRI, PET, cerebrospinal fluid analysis, and many other forms of imaging and biomarker data. These data are publicly accessible to the global scientific community to promote research on AD and related neurodegenerative disorders. The data are stored and shared through a public database.

In the initial phase of constructing the dataset, the focus was primarily on the overall characteristics of all patients with cognitive impairment within the MCI category, without specifically considering the sample ratio between EMCI and LMCI. This approach was adopted to provide a comprehensive understanding of MCI as a whole category of cognitive impairment. To further investigate the subtle differences between EMCI and LMCI and to improve the model's discriminative ability, the decision was made to resample the data within these two subtypes. This adjustment aims to create a more balanced and representative dataset for subsequent analysis, model training, and prediction tasks. In this study, T1-weighted sMRI image data were used, including 350 AD data, 629 MCI data, 350 NC data, 350 EMCI data, and 318 LMCI data. A detailed account of the selected data is provided in Table 1.

**Table 1:** Demographic characteristics and scale scores of the study participants.

Group	AD	MCI	NC	EMCI	LMCI
Male/Female	176/174	384/245	171/179	205/145	142/176
Age	76.99 ± 7.38	76.12 ± 7.36	77.59 ± 5.42	72.04 ± 7.71	73.25 ± 7.56
MMSE	21.49 ± 4.62	25.93 ± 3.52	29.05 ± 1.19	28.51 ± 1.71	26.57 ± 3.47

Age and Mini-Mental State Examination (MMSE) scores are presented as mean±standard deviation.

#### Data preprocessing.

The images downloaded from ADNI have already undergone gradient distortion correction and gradient non-uniformity correction [24]. Building upon this, further preprocessing steps were performed. The first step involved performing anterior commissure-posterior commissure correction on the downloaded DICOM format images. Then, skull stripping was applied to remove non-brain tissue. Finally, the skull-stripped sMRI images were registered to the standard brain atlas of the Montreal Neurological Institute space [25]. The size of the final processed images is 121 × 145 × 121. The dataset was randomly divided into a training set and a test set in the ratio of 7:3, where the training set constitutes 70 percent of the set and the test set constitutes 30 percent of the set.

#### Data augmentation.

To mitigate the risk of overfitting due to the relatively limited dataset, data augmentation techniques were performed to improve classification accuracy [26]. A series of augmentation operations were applied to the preprocessed 3D images using TorchIO in Python. The augmentation operations on the training set included: intensity rescaling, which adjusts the range of pixel values; random affine transformation, which performs operations such as translating, rotating, and scaling; random flipping, which flips the image horizontally, vertically, or along a specific dimension; adding random noise or blurring; random masking, which generates masked regions in the image; and Z-normalization, which normalizes the image data to a distribution with a mean of 0 and a standard deviation of 1. This latter operation helps to speed up training and improves model convergence. The test set underwent only Z-score normalization.

### Methods

#### CNN architecture.

**Fig 2:**
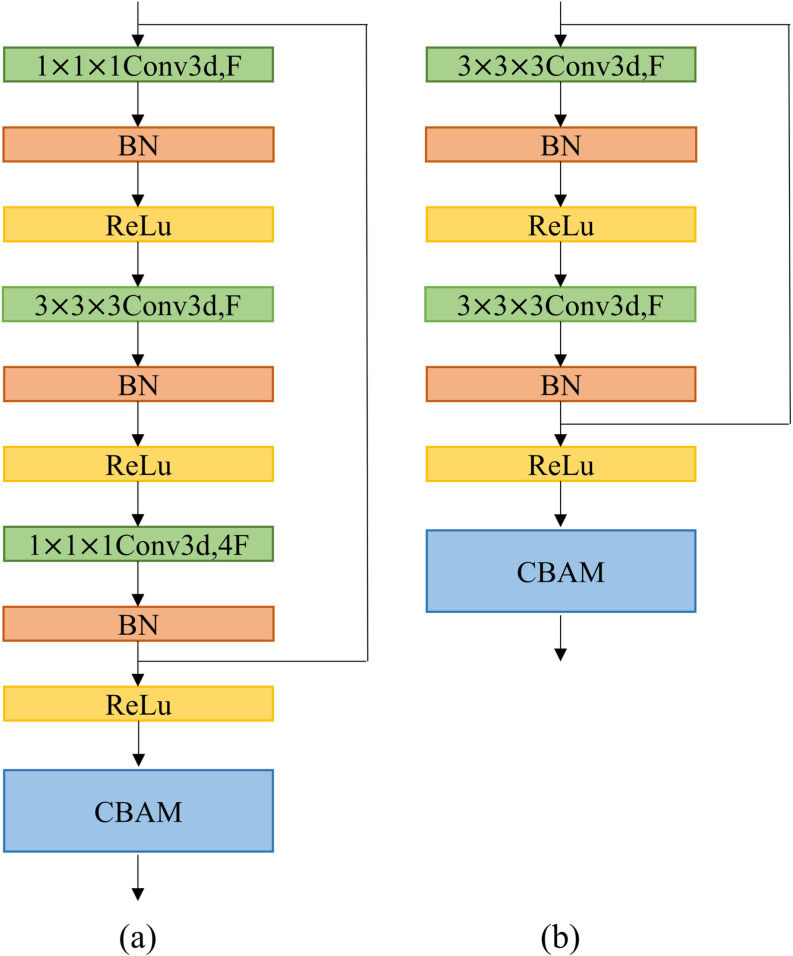
The structure of the bottleneck block and basic block connected to CBAM. (a) illustrates the bottleneck block, while (b) depicts the basic block. *x*_×_*x*_×_*x*Conv3d, F denotes the kernel size and the number of feature maps, respectively, and BN refers to batch normalization.

ResNet is a convolutional neural network proposed by He et al. [12]. The main feature of the ResNet architecture is the introduction of a residual learning mechanism, which effectively solves the problems in deep network training by inter-layer connections and downsampling strategies. The ResNet structure is designed with blocks of different depths, including the basic block and the bottleneck block. ResNet-18 and ResNet-34 are composed of different numbers of basic blocks, while ResNet-50 is constituted of bottleneck blocks. The ResNet architecture uses an average pooling layer and a fully connected layer at the end of the network to transform the convolutional feature maps into the final classification results. In this study, 3D versions of ResNet-18, ResNet-34, and ResNet-50 are used, and they are combined with CBAM for classification. Fig 2 shows the structure of the two blocks integrated with CBAM at the end of the ResNet.

Attention mechanisms have an important role in human perception. Nowadays, numerous researchers have sought to enhance the performance of CNNs by integrating attention mechanisms into their architectures [18,27]. The attention mechanism enables the network to focus on important features while suppressing irrelevant ones, thus enhancing the model's focus and learning ability related to the input data.

The CBAM is a lightweight and generalized module proposed by Woo et al. [28] that can be integrated into any CNN architecture. The CBAM combines both the channel attention mechanism and the spatial attention mechanism, which can improve the performance of the model while maintaining a relatively low computational overhead. In this study, the CBAM has been modified to be compatible with 3D CNN and incorporated at the end of the ResNet. The specific network structure is given in Table 2.

**Table 2:** The structure of the 3D ResNet models.

	3D ResNet-18	3D ResNet-34	3D ResNet-50
	Input		
	7 × 7 × 7Conv3d		
	BN		
	ReLu		
	Max pooling		
Block	Basic	Basic	Bottleneck
Layer 1 (F = 32)	2	3	3
Layer 2 (F = 64)	2	4	4
Layer 3 (F = 128)	2	6	6
Layer 4 (F = 256)	2	3	3
	CBAM		
	Average pooling		
	Fully connected		
	Softmax		

Each ResNet model has a convolutional layer, a BN layer, ReLu, and a max pooling layer before layer 1. Additionally, each model incorporates a CBAM, an average pooling layer, a fully connected layer, and a Softmax layer after layer 4. 1 × 1 × 1 convolutional kernel is used for downsampling in the first block of layer 2, layer 3, and layer 4 of each model.

#### Ensemble learning.

The core idea of the ensemble method is to utilize the classification results of multiple models and combine them in some way to achieve superior classification results than any individual model. This study uses a weighted probability-based fusion method to get the final results [29,30]. Specifically, different weights of 0.4, 0.3, and 0.3 are assigned to the classification results of the three models. The model with the optimal performance is allocated a greater weight, reflecting its superior predictive capabilities, while the other two models are assigned relatively lower weights. This weighting strategy serves to balance the contributions of the best-performing model, ensuring that its strengths are effectively utilized without disproportionately influencing the final output. Simultaneously, it mitigates the risk of excessive reliance on the model with the least favorable performance, thereby preserving the integrity of the ensemble. By taking into account the predictive power of all participating models in the overall integration process, this method enhances the robustness and reliability of the ensemble results.

The weighting strategy (0.4, 0.3, 0.3) employed in this study was determined through systematic pre-experimentation and parameter optimization. Multiple weight combinations were evaluated, such as (0.5, 0.3, 0.2, accuracy: 0.9560), (0.6, 0.2, 0.2, accuracy: 0.9524), and (0.7, 0.2, 0.1, accuracy: 0.9451). The (0.4, 0.3, 0.3) combination achieved the accuracy of 0.9597, demonstrating superior classification performance. Notably, assigning an excessively dominant weight to the best-performing model did not improve overall performance, emphasizing the necessity of a balanced weighting scheme.

Let *W*^*i*^ denote the weight of the *i*-th model. The model's predicted probabilities are represented as


$$\begin{array}{c} {}*20cP^{i}=\left(p_{1}^{i}\cdots p_{n}^{i}\right) \end{array}$$ (1)

where *n* denotes the number of classes, *n* is 2 or 4. Then normalize *P*^*i*^ as


$$\begin{array}{c} \begin{array}{c} {}*20cP^{i}=\frac{P^{i}}{max\left(P^{i}\right)} \end{array} \end{array}$$ (2)

The final class label is determined as


$$\begin{array}{c} {}*20cc=argmax\left(\sum_{i=1}^{3}W^{i}p_{1}^{i}\cdots \sum_{i=1}^{3}W^{i}p_{n}^{i}\right) \end{array}$$ (3)

#### Data partition strategy.

In this study, all preprocessing steps are conducted on the raw data before dataset division. The dataset is randomly split into a training set (70%) and a test set (30%), ensuring each sample is exclusively assigned to one subset to prevent overlap. Data augmentation is applied only to the training set to artificially expand its size and enhance model generalization, while the test set undergoes only necessary normalization procedures and remains untouched during training. The test set is strictly reserved for final evaluation and is never used for model optimization or hyperparameter tuning. This design ensures the test set serves as an independent benchmark, enabling unbiased evaluation of model performance. By maintaining clear separation between training and testing phases, the methodology ensures the reliability and validity of the experimental results.

#### Evaluation metrics.

In the classification tasks, the prediction results are categorized into the following four cases: TP denotes positive samples that have been correctly predicted as positive, TN indicates negative samples that have been correctly predicted as negative, FP refers to negative samples that have been incorrectly predicted as positive, and FN signifies positive samples that have been incorrectly predicted as negative. The evaluation metrics used in this study include accuracy, recall, precision, F1-score, and area under the curve (AUC). In the case of multiclass classification, only the first four evaluation metrics are used.

Accuracy is the ratio of the number of correctly predicted samples to the total number of samples.


$$\begin{array}{c} \begin{array}{c} {}*20cAccuracy=\frac{TP+TN}{TP+FP+FN+TN} \end{array} \end{array}$$ (4)

Recall is the ratio of the number of correctly predicted positive samples to the total number of actual positive samples.


$$\begin{array}{c} \begin{array}{c} {}*20cRecall=\frac{TP}{TP+FN} \end{array} \end{array}$$ (5)

Precision is the ratio of the number of correctly predicted positive samples to the total number of samples that are predicted as positive.


$$\begin{array}{c} \begin{array}{c} {}*20cPrecision=\frac{TP}{TP+FP} \end{array} \end{array}$$ (6)

F1-score is the harmonic mean of precision and recall, which provides a balance between the two metrics by considering both precision and recall.


$$\begin{array}{c} \begin{array}{c} {}*20cF1-score=\frac{2\times Precision\times Recall}{Precision+Recall} \end{array} \end{array}$$ (7)

The AUC is calculated by summing the area under the receiver operating characteristic (ROC) curve. Although the ROC curve may not always lead to a straightforward comparison of different models, the AUC serves as an effective measure of the model's overall performance, with AUC values approaching 1 indicating superior performance.

## Results

In this study, classification tasks focus on the detection of MCI. Initially, binary classification is performed between MCI and NC, as well as between MCI and AD. The state of MCI can be further subdivided into EMCI and LMCI. The distinction between these two states is important for individualized treatment, scientific research, and the optimal allocation of social health resources. Therefore, EMCI and LMCI are classified, and classification is performed across the four states: NC, EMCI, LMCI, and AD.

The experiments were conducted on a computing platform equipped with the Python 3.10.12 and PyTorch 2.0.1 software, utilizing an NVIDIA GeForce RTX 4090 graphics processing unit (GPU) with 64 gigabytes (GB) of memory. The network is trained with a batch size of 4, and a learning rate of 0.0001, and the training consists of 100 epochs.

### Ablation experiments

This section presents results comparing the performance of models with and without CBAM, as well as with and without data augmentation. Taking the classification tasks of MCI and NC as an example, this study trained three 3D ResNet models. First, without data augmentation, the impact of CBAM on the performance of the 3D ResNet models is examined. Table 3 presents the performance differences between models with and without CBAM. The results indicate that incorporating CBAM improves classification performance and accuracy across the three ResNet models. Subsequently, the model with CBAM is employed to investigate the impact of data augmentation on model performance. The term “data augmentation” is represented as “DA” in Table 3. The results in Table 3 indicate that the classification performance of the three ResNet models has been further improved.

**Table 3:** Results of ablation experiments.

MCI vs. NC	Model	Accuracy	AUC	Recall	Precision	F1-score
Without CBAM Without DA	3D ResNet-50	0.8923	0.8157	0.8880	**0.9407**	0.9136
	3D ResNet-34	0.8769	0.8554	0.8720	**0.9316**	0.9008
	3D ResNet-18	0.8718	0.9020	0.8400	**0.9545**	0.8963
With CBAM Without DA	3D ResNet-50	0.9179	0.9014	0.9600	0.9160	0.9375
	3D ResNet-34	0.8974	0.8791	0.9440	0.9008	0.9219
	3D ResNet-18	0.8872	0.8491	**0.9840**	0.8601	0.9179
With CBAM With DA	3D ResNet-50	**0.9231**	**0.9054**	**0.9680**	0.9167	**0.9416**
	3D ResNet-34	**0.9128**	**0.8974**	**0.9520**	0.9154	**0.9333**
	3D ResNet-18	**0.9026**	**0.9086**	0.9120	0.9344	**0.9231**

**Fig 3:**
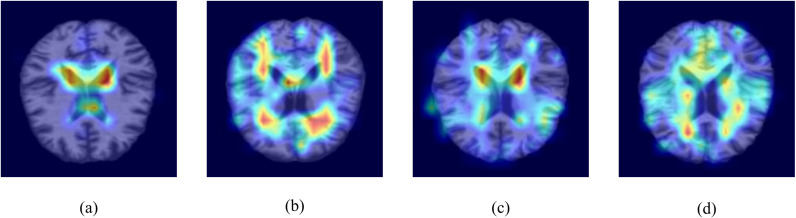
Visualization of CBAM. (a) represents the AD category, (b) represents the NC category, (c) represents the EMCI category, and (d) represents the LMCI category.

In this study, visualization techniques were integrated to illustrate the role of the CBAM mechanism within the neural network. Fig 3 presents MRI cross-sectional slices from four different categories, with each sample processed through this mechanism. In Fig 3, regions highlighted in shades closer to red indicate areas that received higher attention from the model during the classification decision process. The more intense the red hue, the greater the attention the model paid to these regions during classification. The CBAM mechanism enhances the model's focus on these key regions, enabling it to more effectively extract and emphasize pathological features in AD classification. This attention mechanism aids the model in identifying critical abnormalities or features, thereby significantly improving classification accuracy. The visualization provides a more intuitive explanation of the role of the CBAM attention mechanism within the model, offering valuable insights for model optimization and interpretability.

Based on the above results, this study ultimately uses models with CBAM to classify images after performing data augmentation. Taking the classification tasks of MCI and NC as an example, the differences in ensemble performance are discussed. There are four combination methods for the three ResNet models. This study compares pairwise ensemble combinations of the three models with the ensemble of all three models. In Table 4. “Ensemble 1” denotes the ensemble of 3D ResNet-50 and 3D ResNet-18, “Ensemble 2” denotes the ensemble of 3D ResNet-50 and 3D ResNet-34, “Ensemble 3” denotes the ensemble of 3D ResNet-34 and 3D ResNet-18, and “Ensemble” denotes the ensemble of all three networks. Compared to individual models, both “Ensemble 1”, “Ensemble 3” and “Ensemble” showed improved accuracy, while Ensemble 2 did not exhibit an accuracy increase. However, the AUC values for all three methods were enhanced, indicating an overall improvement in performance. The results indicate that the best performance is achieved when using the ensemble of three models.

**Table 4:** Ensemble results of different models.

MCI vs. NC	Accuracy	AUC	Recall	Precision	F1-score
Ensemble 1	0.9385	0.9520	0.9440	**0.9593**	0.9516
Ensemble 2	0.9231	0.9553	0.9680	0.9167	0.9416
Ensemble 3	0.9333	0.9559	0.9600	0.9375	0.9486
Ensemble	**0.9487**	**0.9689**	**0.9840**	0.9389	**0.9609**

### All classification results

Based on the aforementioned results, this study employed the proposed method for binary classification between MCI and AD, as well as between EMCI and LMCI. Additionally, a four-class classification involving NC, EMCI, LMCI, and MCI was performed. The results are presented in Tables 5 and 6.

**Table 5:** Binary classification results.

Binary	Model	Accuracy	AUC	Recall	Precision	F1-score
MCI vs. NC	3D ResNet-50	0.9231	0.9054	0.9680	0.9167	0.9416
	3D ResNet-34	0.9128	0.8974	0.9520	0.9154	0.9333
	3D ResNet-18	0.9026	0.9086	0.9120	0.9344	0.9231
	Ensemble	**0.9487**	**0.9689**	**0.9840**	**0.9389**	**0.9609**
MCI vs. AD	3D ResNet-50	0.9128	0.8849	0.9840	0.8913	0.9354
	3D ResNet-34	0.9026	0.8863	0.9440	0.9077	0.9255
	3D ResNet-18	0.8974	0.8854	0.9280	**0.9134**	0.9206
	Ensemble	**0.9231**	**0.9289**	**0.9760**	0.9104	**0.9421**
EMCI vs. LMCI	3D ResNet-50	0.9323	0.9310	0.9048	0.9500	0.9268
	3D ResNet-34	0.9474	0.9468	**0.9365**	0.9516	0.9440
	3D ResNet-18	0.9173	0.9302	0.8571	0.9643	0.9076
	Ensemble	**0.9549**	**0.9645**	0.9206	**0.9831**	**0.9508**

**Table 6:** Multiclass classification results.

Multiclass	Accuracy	Recall	Precision	F1-score
3D ResNet-50	0.9267	0.9270	0.9268	0.9269
3D ResNet-34	0.9158	0.9171	0.9179	0.9175
3D ResNet-18	0.9451	0.9452	0.9448	0.9450
Ensemble	**0.9597**	**0.9599**	**0.9596**	**0.9597**

**Fig 4:**
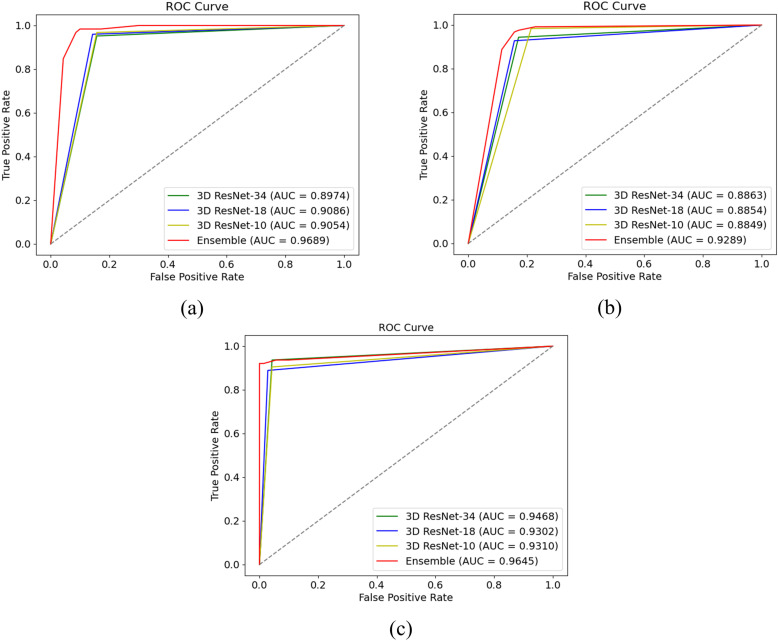
The ROC curve of binary classification. (a) shows the ROC curve of MCI vs. NC, (b) shows the ROC curve of MCI vs. AD, and (c) shows the ROC curve of EMCI vs. LMCI.

**Fig 5:**
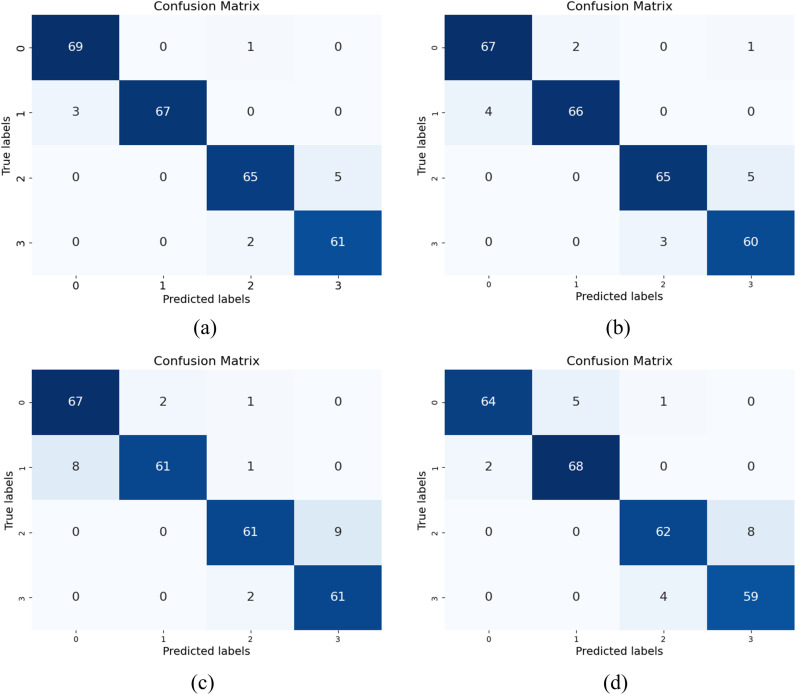
Confusion matrix of multiclass classification results. (a) is the confusion matrix of the final ensemble results, (b) is the confusion matrix of the 3D ResNet-18 classification results, (c) is the confusion matrix of the 3D ResNet-34 classification results, and (d) is the confusion matrix of the 3D ResNet-50 classification results.

Furthermore, Figs 4 and 5 present the ROC curve for binary classification and the confusion matrix for four-class classification, respectively. The data presented in the tables demonstrate that, across all classification tasks, ensemble learning achieves a notable improvement compared to individual models. The ROC curve for the binary classification task clearly illustrates the superior performance of ensemble learning relative to a single model.

## Discussion

This section compares the results of this study with those of previous studies, then points out the limitations of the proposed method and discusses potential approaches to address these limitations in future research.

The results obtained in this study indicate that the proposed method is a viable approach for AD classification, yielding superior results. To better illustrate the effectiveness of the method, a comparison with prior work was conducted. The results are presented in Tables 7 and 8. Data from Tables 7 and 8 demonstrate that the method outperforms others in both binary and four-class classification tasks.

**Table 7:** Comparison of binary classification results.

Binary	References	Accuracy	AUC	Recall	Precision	F1-score
MCI vs. NC	[17]	0.8085	0.8507	0.7733	–	–
	[31]	0.6225	–	0.5979	0.6170	0.6073
	[32]	0.7620	0.7750	0.7950	–	–
	[33]	0.9260	0.9422	–	–	0.9273
	[34]	0.7663	0.8270	0.7844	–	–
	**Ours**	**0.9487**	**0.9689**	**0.9840**	**0.9389**	**0.9609**
MCI vs. AD	[17]	0.8734	0.9073	0.8450	–	–
	[25]	0.9120	–	0.8110	0.9090	0.8570
	[31]	0.7170	–	0.7021	0.7174	0.7097
	[33]	0.9135	0.9224	–	–	0.9107
	[34]	0.8429	0.8150	0.7862	–	–
	**Ours**	**0.9231**	**0.9289**	**0.9760**	**0.9104**	**0.9421**
EMCI vs. LMCI	[35]	0.8409	0.8889	0.8235	–	0.8529
	[36]	0.7000	0.7900	0.6500	0.8300	0.6600
	[37]	0.9422	0.9422	0.9408	–	–
	[38]	0.8085	0.8487	0.7234	0.6344	–
	[39]	0.7710	–	**0.9608**	0.7543	0.8593
	**Ours**	**0.9549**	**0.9645**	0.9206	**0.9831**	**0.9508**

**Table 8:** Comparison of multiclass classification results.

Multiclass	References	Accuracy
NC vs. EMCI vs. LMCI vs. AD	[30]	0.8333 ADNI
	[37]	0.9388 ADNI+ Local dataset
	[40]	0.9183 ADNI 0.9385 OASIS
	[41]	0.9300 ADNI
	**Ours**	**0.9597** ADNI

Accuracy is indicated with the data source. OASIS refers to the Open Access Series of Imaging Studies, while the local dataset represents data obtained from local hospitals.

Several studies have used 3D CNN architectures for classification tasks [17,25,31,34,39,41]. The observed performance differences among these architectures are influenced by the datasets employed and the specific training methodologies adopted. Notably, some studies have indicated that 3D CNN architectures outperform 2D CNN architectures in terms of classification performance [17,31]. Liu et al. [32] proposed a multi-model deep learning framework. Their results show that multi-model approaches yield better performance compared to single models. Furthermore, several studies implemented ensemble learning techniques, which demonstrate superior performance [36,37,39]. Variations in datasets, model architectures, and the specific ensemble strategies employed may contribute to the discrepancies in results. Although the outcomes of other studies may fall short of our method, they provide valuable considerations for future research avenues.

In this study, 3D images are used as inputs to the network. The approach was based on 3D ResNet-18, 3D ResNet-34, and 3D ResNet-50, which are combined with CBAM for AD classification. The classification results of the three networks are integrated through a weighted probability-based ensemble method to get the final results. It is well known that 3D whole-brain images contain rich information, but the rich image information contains not only critical information for classification but also some unimportant information. Accordingly, this study combines the network with the attention mechanism to focus the CNN attention on the key information related to the classification tasks. The results of our study show that the attention mechanism helps improve the model's performance. Meanwhile, the limited amount of data can affect classification performance. Therefore, this study employs data augmentation to address this issue, and the previous results have demonstrated the effectiveness of this approach.

A single CNN may be limited by factors such as insufficient data and the choice of model structure, potentially resulting in suboptimal classification performance. To overcome these limitations, ensemble learning becomes an effective strategy. It can combine the results of multiple CNNs, thus reducing the bias of a single model, significantly improving the performance of classification tasks, and enhancing the stability of the model for superior performance in practical applications. Unlike simple majority voting methods, the ensemble method this study adopts relies on the probability distributions of each model's output. Specifically, different weights are assigned to each model based on its classification accuracy, and then the weighted probabilities are summed to obtain the final integrated probability for prediction. This method effectively improves the accuracy and reliability of classification by considering the prediction confidence of each model. The experimental results clearly show that this weighted probability-based ensemble method performs better than single CNN models.

Although the research method achieved good results in AD classification, there are still some limitations that, if addressed, could further optimize the network's performance and generalization ability. The first is that this study only used a single modality, sMRI images, for training. Future work could consider combining other modality images, such as PET, and applying multimodal data for AD classification. Secondly, the ensemble method this study proposed uses different ResNet models. Future research could investigate the effects of integrating other network models, and explore different weighting strategies for the models in the ensemble method.

## Conclusion

The accurate diagnosis of MCI in the clinic is particularly important, as it not only influences the treatment plans for patients but also relates to the prevention and management of future cognitive decline. To this end, this study proposes an innovative method aimed at classifying different cognitive states using 3D sMRI data. These states include classifying MCI and NC, MCI and AD, EMCI, and LMCI, as well as NC, EMCI, LMCI, and AD. Methodologically, 3D whole-brain images are used as inputs to three 3D ResNet models, incorporating the CBAM attention module to enhance feature representation. This attention module plays a crucial role in improving the model's ability to focus on relevant features. To further improve the model's generalization ability and robustness, data augmentation techniques are applied. Ultimately, the classification results of the three models are integrated using a weighted probability ensemble method to achieve more accurate final classifications. The experimental results demonstrate that the proposed method exhibits high effectiveness and accuracy in predicting different cognitive states, thereby validating the model's performance and providing a solid foundation for future clinical applications. This study provides potential support for interventions and treatments for AD and its precursor stages.

## Supporting information

S1 File Data-related information. (ZIP)
